# Bursting of the upper jaw prosthesis and fractures of the lower jaw as indirect injury pattern caused by a headshot: a case report

**DOI:** 10.1007/s12024-023-00710-6

**Published:** 2023-09-08

**Authors:** Anja Weber, Claudia Wöss, Beat P. Kneubuehl, Walter Rabl

**Affiliations:** 1 Institute of Legal Medicine, Medical University of Innsbruck, Müllerstraße 44, 6020 Innsbruck, Austria; 2 Bpk Consultancy GmbH, Thun, Switzerland

**Keywords:** Suicide, Gunshot, Dental prosthesis, Autopsy, Temporary cavity, Ballistics

## Abstract

Gunshots to the human body can cause direct and indirect injuries. Direct injuries are a consequence of the projectile guiding its way through the body, creating a permanent wound channel and thereby damaging the penetrated as well as the adjacent tissue. In addition, the temporary wound cavity is responsible for indirect injuries occurring distant to the actual wound tract. This can potentially affect different types of tissue, like blood vessels, organs, or bones, that are not directly passed through by the projectile. For this case report, we describe a suicidal headshot to the temporal area where the extension of the temporary wound cavity and its subsequent collapse led to massive energy transfer to the surrounding tissue leading to breakage of the upper dental prosthesis and fractures of the lower jaw. Thereby outlining the ballistic mechanisms causing indirect injury pattern that have to be considered when examining gunshot wounds.

## Introduction

The second most common way to commit suicide in Austria besides hanging is using a shotgun or rifle (20%). Thereby, suicidal headshots are frequent. Common bullet entry areas are, for example, the temporal area and the mouth. In men, the general risk to commit suicide increases with age [1]. As people get older, also the number of those wearing dental prostheses increases, which might get damaged during such incidences. While damage of prostheses due to a shot in the mouth is expectable, shots to other areas of the head leading to breakage or even blasting of those have, unless to our knowledge, not yet been published.

## Casuistic

**Fig. 1:**
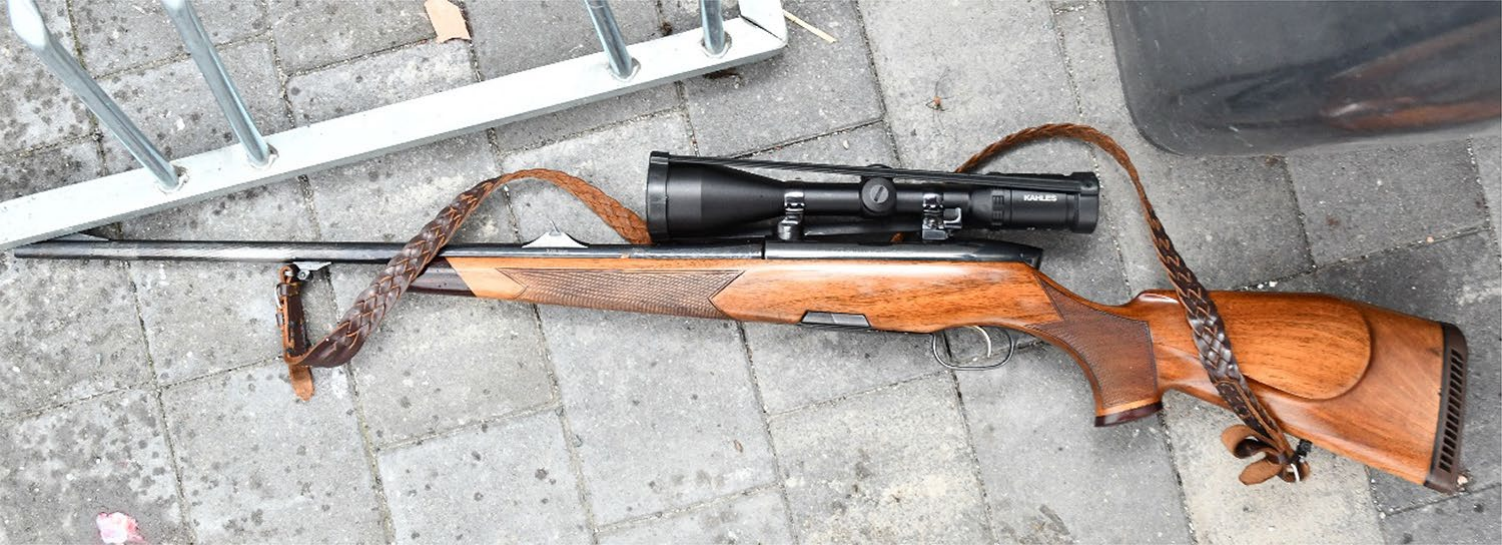
Rifle found near the body

A 77-year-old man was found dead in the front of a house. A hunting rifle with a total length of 1115 mm (caliber 270 Winchester, Steyr Arms GmbH, Model Luxus) was found near the body (Fig. 1).

**Fig. 2:**
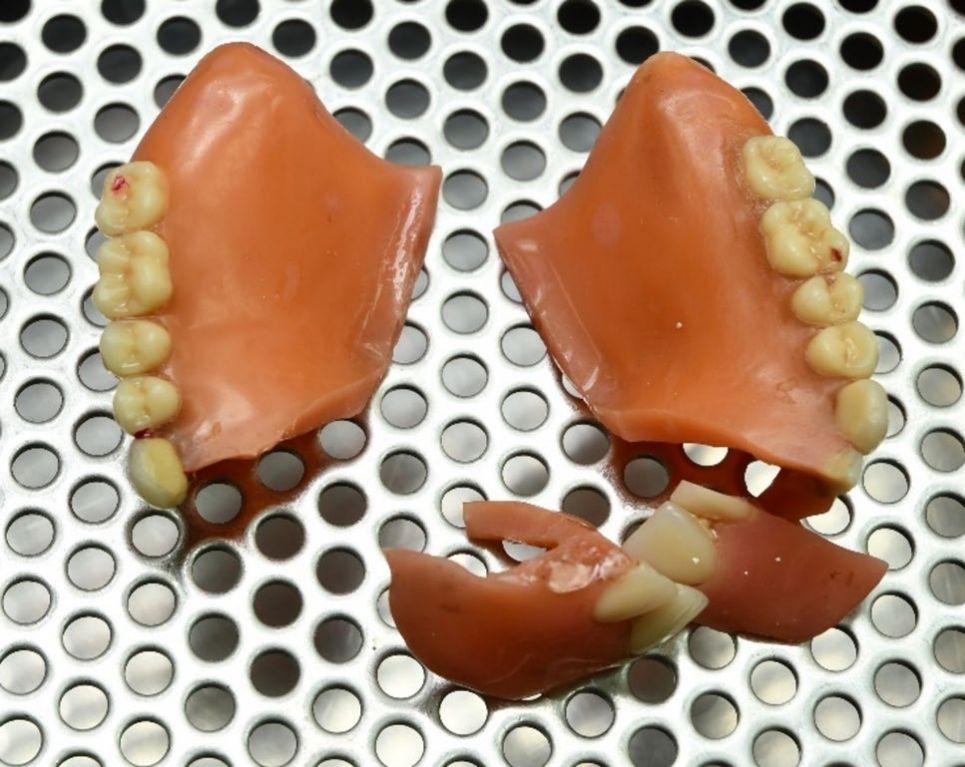
Broken upper jaw prosthesis

The scenery showed multiple blood spots, with a big pool around the men’s head and traces of tissue spread all over the area around the body. The skull was shattered with evisceration of the brain and laceration of the face. On first impression, the police assumed a shot to the mouth, due to no obvious bullet entry area. During autopsy, the bullet entry wound could be located at the left temporal area and therefore a mouth shot could be excluded. Nevertheless, a surprising discovery has been found in the oral cavity: The man has been wearing an upper and lower jaw dental prosthesis. The upper prosthesis has been shattered to four pieces, one break went through the middle line and two breakage areas were located in the front teeth area (Fig. 2). One part of the broken prosthesis has been found at the scenery located next to the man’s feet, and the three other parts remained inside the mouth and were extracted during autopsy. The maxilla itself was movable by means of a Le Fort I fracture.

**Fig. 3:**
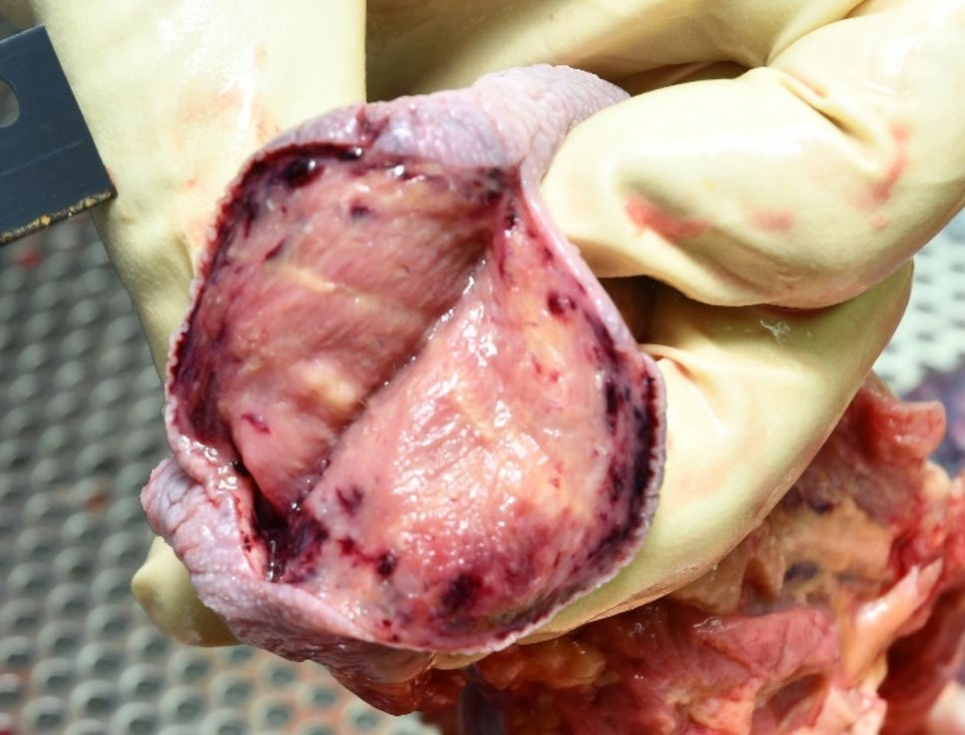
Squeezed edges of the tongue

The lower jaw prosthesis was intact and lied above the head. The lower jaw bone showed two fractures, one on each side. Additionally, the edges of the tongue were visibly squeezed as a result of the crashing dental jaw prostheses of upper and lower jaw (Fig. 3). These autopsy results led to the assumption that massive indirect forces had been involved. The overall result of the autopsy could confirm a suicidal shot to the left temporal area with no signs of other significant injuries than those caused direct or indirect by the shot. Toxicological analysis of blood and urine revealed no significant impairment at the time of death. Cause of death was therefore declared as a consequence of the headshot leading to blasting of the skull with evisceration of the brain and massive loss of blood.

## Ballistic background

Depending on the individual ammunition used, the burning of the powder of a cartridge in the caliber 270 Win. results in 2.8–3.2 l of gas. This amount of gas exits with a muzzle pressure of 600–700 bar. Furthermore, the muzzle velocity for such bullets is 870–1040 m/s and the discharging velocity of the gas, following straight after the muzzle leaving bullet into the forming shot channel, reaches up to 1000–1120 m/s. In combination with the energy resulting from the deceleration of the bullet at the men’s head, a massive pressure surge runs through the temporary wound cavity. Therefore, the high level of destruction is conclusive, especially when considering that the temporary wound cavity is additionally bloated with about 3 l of gas.

## Discussion

The most important concept in the ballistic field of higher-velocity bullets is that of the temporary cavity. As the formation of the temporary cavity creates a wound in living beings, it is more accurate to speak of a “temporary wound channel” [2].

A projectile guiding its way through the body causes a permanent wound channel as well as a temporary wound cavity, whereas both play an important role to understand the mechanism of injury. The high energy impact that is transferred to the channel surrounding tissue leads to pressure waves that displace tissue, whereas a vacuum is generated, before the temporary cavity collapses and tissue bounces back to its initial position. This phenomenon repeats until no further energy remains, but each time to a lesser extent, like a ball that has been thrown onto the floor and is bouncing with decreasing height until it stops, thus causing the tissue to oscillate [3, 4]. This kind of indirect injury, for example, indirect fractures, caused by a gunshot is already known to occur at different areas of the body. Kieser et al., for example, examined the mechanism of indirect femoral fractures, caused by a projectile passing close to the bone without direct contact. The objective of the study was to determine whether the main cause of injury is the expansion, collapse, or oscillation of the temporary cavity. It could be demonstrated that the cause of fracture is the radial displacement of soft tissue material by the expansion of the temporary cavity and was related to the proximity of the expansion area to the bone [5].

Furthermore, Zhang et al. studied the biomechanisms of ballistic brain injury via high-speed digital video photography and captured pressure data in a transparent brain simulant to understand the mechanisms of the temporary wound cavity. Testing two different projectile sizes, they could outline that the size of the temporary cavity is dependent on the size of the projectile and larger projectiles with higher velocity introduce larger and longer—lasting temporary cavities. For these kinds of projectiles, also the pressure differences between center and boundary location were higher, thereby contributing to the extent of injury. While capturing pressure data, they could show a first high peak pressure corresponding to the entry of the projectile followed by a pressure change as the projectile exited the simulant, corresponding to the pulsation of the temporary cavity. A positive peak pressure with negative pressure in the surrounding area was determined at the time the temporary cavity collapsed. A negative peak pressure could be registered at the interval between maximum expansion of the temporary cavity and its collapse. Subsequently, they could demonstrate variations in pressure gradients depending on the location [6].

In the described case, a large cartridge was applied, which is commonly used for the hunting of light to middle heavy game animals. Following the results of Zhang et al., one can implicate that firing such large projectiles, with a muzzle energy range of 3300–3700 Joule (depending on the type of bullet), can lead to the formation of a large and long-lasting temporary cavity with high degree of soft tissue displacement. In the case of a headshot, where the skull is a natural barrier with less elasticity and capacity to deform, compared to soft tissue, the transferred energy possibly causes fractures when the elastic limit is exceeded, especially in close proximity to the temporary cavity, as the study of Kieser et al. outlined. According to Di Maio, the critical muzzle velocity for hunting bullets to cause bursting of organs is 457–610 m/s, which is—as mentioned above—with 870–1040 m/s for the described case more than exceeded [7].

Overall, these mechanisms taken together with the ballistic background of the described case can feasibly explain the bursting of the upper jaw prosthesis and fracturing of the lower jaw, which underlines the potential impact of the temporary wound cavity leading to massive indirect injuries.

## Key points

1. The most important concept in the ballistic field of higher-velocity bullets is that of the temporary cavity.
2. The permanent and the temporary wound cavity both play an important role for the mechanism of injury.
3. Firing large projectiles, the formation of a temporary cavity can lead to massive tissue displacement, which potentially causes fractures when the elastic limit of tissue is exceeded.
4. The expansion of the temporary cavity can lead to massive energy transfer and thereby injure body parts that have not been in direct contact with the fired bullet.

## Data Availability

Due to data protection regulations, data is not publicly available.
